# Factors associated with medication adherence among adult diabetic patients of Bangladesh

**DOI:** 10.1371/journal.pone.0343647

**Published:** 2026-09-17

**Authors:** Simanta Roy, Mohammad Azmain Iktidar, Mowshomi Mannan Liza, Madhu Ritu Bhadra Medha, Tonmoy Chowdhury, Nasrat Khanam Nila, Arpita Biswangree, Irfat Islam Eva, Nishat Tasnim, Pratik Kumar Banik, Shourov Barua, Mahzabeen Islam, Sreshtha Chowdhury, Gladys Ibanez, Mohammad Delwer Hossain Hawlader

**Affiliations:** 1 Department of Epidemiology, Robert Stempel College of Public Health & Social Work, Miami, Florida, United States of America; 2 Department of Public Health, North South University, Dhaka-1229, Bangladesh; 3 School of Research, Chattogram, Bangladesh; 4 Public Health Promotion and Development Society (PPDS), Dhaka, Bangladesh; 5 Rangamati Medical College, Hospital Road, Rangamati, Bangladesh; 6 Comilla Medical College and Hospital, Dr Akhtar Hameed Khan Rd, Kuchaitoli, Cumilla, Bangladesh; 7 Chittagong Medical College, Chittagong, Bangladesh; 8 Toxicology Society of Bangladesh, Chattogram, Bangladesh; 9 American International University-Bangladesh (AIUB), Kuratoli, Khilkhet, Dhaka, Bangladesh; 10 NSU Global Health Institute (NGHI), North South University, Dhaka, Bangladesh; Al Azhar University Gaza, PALESTINE, STATE OF

## Abstract

**Introduction:**

Medication non-adherence remains a considerable public health concern among diabetic patients in Bangladesh. It can lead to waste of medication, disease progression, a lower quality of life, and increased use of medical resources. The aim of this study is to comprehensively assess the factors associated with medication adherence among diabetic patients in Bangladesh.

**Materials and methods:**

A sample of 426 diagnosed diabetic patients was conveniently recruited from government hospitals (secondary and tertiary level), outpatient clinics, and physicians’ private practices between August and September 2023. A pretested semi-structured questionnaire was employed for interviewer-administered data collection. The questionnaire included socio-demographic history, personal characteristics, disease-related information, and a medication adherence rating scale (MARS-5). A multivariable logistic regression model was used to explore the factors associated with medication adherence.

**Results:**

The mean age of the participants was 55.49±10.19 years, and half of them were female (50.00%) and lived with a partner (50.70%). The prevalence of poor medication adherence was 42.25% (95% CI: 37.63–47.01%). Participants who lived with a partner (AOR: 1.66, CI: 1.03–2.68), had higher educational status (AOR: 2.65, CI: 1.08–6.54), understanding of all current medication (AOR: 1.69, CI: 1.04–2.77), satisfaction with healthcare (AOR: 2.53, CI: 1.32–4.88) were associated with good medication adherence. Conversely, a higher number of medications (AOR: 0.87, CI: 0.76–1.00), self-medication (AOR: 0.35, CI: 0.18–0.70), and poor mental health (AOR: 0.53, CI: 0.30–0.92) were associated with a lower likelihood of good adherence.

**Conclusions:**

Medication non-adherence among diabetic patients is considered to be highly prevalent in Bangladesh. Several factors, including educational status, understanding of medications, satisfaction with healthcare, self-medication, mental health, and number of medications, were associated with medication adherence.

## Introduction

Medication adherence is defined by the World Health Organization (WHO) as “the degree to which the person’s behavior corresponds with the agreed recommendations from a health care provider” [1]. Medication non-adherence is a global concern impacting society, patients, and healthcare providers. It is one of the primary factors contributing to the discrepancies between clinical trial results and real-world outcomes [2,3]. This issue is rapidly growing among individuals with chronic illnesses, with an approximate 50% prevalence of medication non-adherence worldwide [4]. Medication non-adherence leads to poor outcomes, increasing healthcare service utilization and costs [5]. Diabetes mellitus (DM) is one of the most common chronic diseases globally, with a significant proportion (80%) of affected individuals residing in low and middle-income countries (LMICs). Previous studies indicate that over half (53–67%) of diabetic patients were non-adherent to their oral medication [6], with an even higher rate inLMICs such as Bangladesh (74%) [7].

Medication adherence involves several critical steps that patients must navigate, including filling, initiating, continuing, and taking the prescription as directed. A systematic review of 98 articles identified various personal and healthcare factors, including age, race, health beliefs, medication costs, and co-pays, significantly impacting medication adherence [8]. However, these factors differ in LMICs due to variations in healthcare systems. For example, in Bangladesh, the regulation of prescription medication sales is inadequate [7], enabling users to self-medicate, which may lead to increased medication consumption [9], and drug-drug interactions potentially impacting medication adherence [10]. This, in turn, can result in inadequate management, increased medication burden, and greater susceptibility to long-term complications like nephropathy, neuropathy, retinopathy, and cardiovascular diseases [11].

Previous research on DM patients in Bangladesh identified only a few socioeconomic factors, e.g., male gender and low annual family income, as factors linked to poor medication adherence [12,13]. Currently, there is a lack of comprehensive data regarding medication and disease-related factors and their impact on medication adherence among DM patients in Bangladesh. For example, an increased number of medications with intricate dosing regimens can affect medication adherence, considering that most DM patients are elderly [14]. For these people, a better understanding of the prescribed medications and their dosing is essential for appropriate use. Prescribers are also crucial in the process, as they are responsible for selecting the most suitable medication and dosage. Patient-physician interaction and communication during prescribing also influence medication adherence [15]. However, no studies to date have examined the role of these factors on medication adherence in Bangladesh.

While there is growing evidence regarding non-adherence to diabetic medications and their consequences in high-income countries [16], detailed information on medication adherence among DM patients in Bangladesh remains limited. A comprehensive analysis of the factors influencing medication adherence is crucial for designing effective strategies to tackle current issues related to medication non-adherence in LMICs like Bangladesh. Therefore, this study aims to evaluate the personal and medication-related factors influencing medication adherence in DM patients in Bangladesh.

## Methods

### Study design and participants

This cross-sectional study was conducted among 426 DM patients in Bangladesh between 01 August and 30 September 2023. Patients with diagnosed DM (according to the National Guideline on Diabetes Mellitus in Bangladesh [17], currently taking diabetic medications, and aged more than 18, were included. Any patients who were foreign nationals, under 18 years old, and currently hospitalized were excluded from the study. The participants were recruited using convenience sampling from ten selected government hospital (secondary and tertiary level), outpatient clinics (six from rural and four from urban areas), and private chambers (five from rural and five from urban areas) of physicians across Bangladesh, which included patients from diverse backgrounds and regions. (Please see supplementary Table 1 in S2 File for details)

According to the Bangladesh Bureau of Statistics (BBS), Urban areas were the locations within city corporations or municipalities where population density is high and have established healthcare infrastructure. However, rural areas were mentioned as outside city corporation or municipality boundaries, including upazila-level and peripheral areas [17].

### Sample size

We initially calculated the sample size (376) considering the sample size formula for single proportions: n = z2 × p × (1 − p)/d2, where: z = 1.96 for a confidence level of 95%, p = proportion (from Islam et al, 2021 [13] =40%), d = margin of error = 0.05. Considering the 10% non-response, the final sample size was 376 + 37.6 = 413.6.

Since we employed convenient sampling, we approached 450 participants, which is more than the required sample size to increase precision and generalizability. Out of 450 participants, 426 (~94.7%) finally completed the survey and were considered for analysis.

## Measures

The questionnaire included socio-demographic history: age, gender (male, female), marital status (currently married, currently not married), family income(in bdt), residence(rural, sub-urban, urban), educational status (no formal education, primary, high school, undergraduate, postgraduate), and personal characteristics: BMI(calculated from self-reported height and weight data), smoking status (current smoker, never smoker, ex-smoker), family history of chronic disease, disability (visual, movement, auditory), self-reported physical health status (good, poor) and self-perceived mental health status (adapted from Mawani & Gilmour (2010) [18], participants were asked “would you say your mental health is: excellent?very good? good? fair? poor?”; the responses were dichotomized: fair/poor as poor and good/very good/excellent as good). It also included disease-related variables: type (type 1, type 2), duration of DM (in months), type of medication used (oral, injectable), dosing of anti-diabetic medication (once/daily, twice/daily, thrice/daily, four times/daily), other chronic diseases (hypertension, chronic obstructive pulmonary disease, chronic kidney disease, ischemic heart disease, cancer, chronic liver disease, and stroke), number of current medications, and understanding of ongoing medication (participants’ comprehension regarding the indications of their currently prescribed medications). To assess the practice of self-medication, the respondents were asked to report the anti-diabetic medications they take on his/her own initiative or on the advice of another person, without consulting a doctor. Moreover, the questionnaire also included the patient’s recommendations for improving medication adherence. This section included four options (discussing treatment options with physicians, frequently revising treatment regimes, paying more attention to mental health while prescribing, and considering patients’ preferences), with a last option as ‘others’ to write any other recommendations they had. Self-reported medication adherence was assessed using a five-item validated tool: Medication Adherence Report Scale (MARS-5) [19,20]. The Cronbach alpha value for the scale was 0.75. The MARS-5 has two behavior non-adherence dimensions: nonintentional (forgetting) and intentional (stopping and skipping doses, changing the dose, and taking a lower dose than prescribed). The respondents rate how often they behave as described by the various statements (I forget to take my medicines, I alter the dose of my medicines, I stop taking my medicines for a while, I decide to skip a dose, I take less than instructed) on a 5-point Likert scale (1  =  always, 5  =  never). Scores for each item were summed to give a total score (range 5−25), with higher scores indicating higher levels of adherence; a total score of 5−21 was considered poor adherence, and a score of 22−25 was good adherence according to Uhlig et al. (2024) and Margolis et al. (2020) [21,22]. The questionnaire was prepared in English and was translated into Bengali with the help of a bilingual (Bengali and English) expert. However, in terms of the MARS-5 scale, we used the ‘backward and forward translation’ approach to translate this validated scale.

### Pretesting

Twenty participants from government and private facilities each participated in a pretest to determine the feasibility and validity of the questionnaire. Necessary changes were made according to the feedback of the participants. The validated scales used in the questionnaire remained unchanged, and only minor language modifications were made.

### Data collection and instruments

The pretested semi-structured questionnaire was inputted into Google Forms and was used for in-person data collection; mandatory items were highlighted with a red asterisk, and a relevant non-response option was available. Trained research assistants approached all participants present in the study sites and described the research and data confidentiality in detail. Once the individuals met the inclusion criteria and consented to voluntary participation in the study, an in-person interview was conducted by the research assistants in the isolated areas of the study sites, maintaining adequate privacy. The average time to complete the interview was ten minutes.

### Statistical analysis

We used Stata (version 17) for data analysis. Histogram, Q-Q plot, and the Shapiro-Wilk test were used to check for normality in continuous data. The mean and standard deviation were reported for quantitative data, whereas frequency with proportion was used to report categorical data. The outcomes were categorized into two groups (poor vs good medication adherence). The association between medication adherence and categorical independent variables was assessed using the chi-square test, and the relationship between adherence and continuous variables was evaluated by an independent sample t-test. Multivariable logistic regression models were fitted to investigate the predictors of medication adherence after including relevant variables from the literature review and significant variables from bivariate analysis. The lowest values of the Akaike Information Criterion and the Bayesian Information Criterion (BIC) were considered while considering the model selection. The variance inflation factor (VIF) was used to measure the presence of multicollinearity (VIF < 5 for all). Complete case analysis was considered for regression analysis. Lastly, the logistic regression analysis obtained adjusted ORs (AORs) and corresponding 95% CIs. Statistical significance was set at α < 0.05.

### Ethics

The Institutional Review Board of North South University approved the research (Approval no: 2023/OR-NSU/IRB/0308), and all participants provided informed written consent. Wherever feasible, the 1964 Declaration of Helsinki and later modifications, latest on October 2024 and comparable ethical standards were followed. Data collection was voluntary, and no incentives were offered to participants. Data was only accessible to the research team and were not disclosed anywhere.

## Results

A total of 426 DM patients were included in this study, and the prevalence of poor and good adherence was 42.25% (CI: 37.63–47.01%) and 57.75% (CI: 52.99–62.37), respectively. According to Table 1, the mean age of the participants was 55.49 ± 10.19 years, with a mean BMI of 25.51 ± 3.87. More than a third of the participants studied up to high school (36.38%) and belonged to urban areas (63.38%). Nearly half of the participants (50.00%) were female and lived with a partner (50.70%). 61.03% reported the presence of other chronic diseases, 53.99% had a family history of chronic diseases, and 15.26% reported some sort of disability (auditory/visual or movement).

**Table 1:** Sociodemographic characteristics and disease-related factors and their relation to medication adherence (N = 426).

Characteristics	Categories	Overall	Poor adherence	Good adherence	*p*-value
		N(%)	N(%)	N(%)	
**Sociodemographic characteristics**					
**Age** (in years), mean (SD)		55.49 (10.19)	54.43 (10.09)	56.27 (10.21)	0.07^a^
**Sex**	Female	213 (50.00)	98 (46.01)	115 (53.99)	0.12^b^
	Male	213 (50.00)	82 (38.50)	131 (61.50)	
**Family income** (in BDT), mean (SD)		52112.45 (57804)	47900 (56311.64)	55330.55 (58836.3)	0.90^a^
**Companionship status**	Doesn’t live with a partner	210 (49.30)	108 (51.43)	102 (48.57)	**<0.001** ^b^
	Lives with partner	216 (50.70)	72 (33.33)	144 (66.67)	
**Educational status**	No formal education	40 (9.39)	21 (52.50)	19 (47.50)	0.14^b^
	Primary	54 (12.68)	27 (50.00)	27 (50.00)	
	High school	155 (36.38)	69 (44.52)	86 (55.48)	
	Undergraduate	128 (30.05)	44 (34.38)	84 (65.63)	
	Postgraduate	49 (11.50)	19 (38.78)	30 (61.22)	
**Region**	Rural	156 (36.62)	70 (44.87)	86 (55.13)	0.41^b^
	Urban	270 (63.38)	110 (40.74)	160 (59.26)	
**Smoking**	Regular smoker	33 (7.75)	12 (36.36)	21 (63.64)	0.30^b^
	Former smoker	72 (16.90)	21 (29.17)	51 (70.83)	
	Never smoker	299 (70.19)	79 (26.42)	220 (73.58)	
	Occasional smoker	22 (5.16)	3 (13.64)	19 (86.36)	
**Alcohol**	Never drinker	396 (92.96)	105 (91.30)	10 (8.70)	0.42^b^
	Occasional drinker	30 (7.04)	291 (93.57)	20 (6.43)	
**Presence of other chronic diseases**	No	166 (38.97)	67 (40.36)	99 (59.64)	0.53^b^
	Yes	260 (61.03)	113 (43.46)	147 (56.54)	
**Disability(visual, movement, auditory)**	No	361 (84.74)	143 (39.61)	218 (60.39)	**0.009** ^b^
	Yes	65 (15.26)	37 (56.92)	28 (43.08)	
**Self-perceived mental health status**	Good	314 (73.71)	110 (35.03)	204 (64.97)	**<0.001** ^b^
	Poor	112 (26.29)	70 (62.50)	42 (37.50)	
**BMI**, mean (SD)		25.51 (3.87)	25.60 (3.73)	25.44 (3.97)	0.66^a^
**Family history of chronic diseases**		230 (53.99)	91 (39.57)	139 (60.43)	0.22^b^
**Disease-related factors**					
**Duration of DM** (in months), mean (SD)		73.28 (75.67)	54.79 (60.26)	86.82 (82.72)	**<0.001** ^a^
**DM medication dosage**	Three times/day	122 (28.64)	63 (51.64)	59 (48.36)	0.09^b^
	Once/day	257 (60.33)	99 (38.52)	158 (61.48)	
	Twice/day	43 (10.09)	16 (37.21)	27 (62.79)	
	Four times/day	4 (0.94)	2 (50.00)	2 (50.00)	
**DM medication type**	Only Oral	289 (67.84)	129 (44.64)	160 (55.36)	0.04^b^
	Only Injectable	64 (15.02)	30 (46.88)	85 (53.13)	
	Both Oral and Injectable	72 (16.90)	21 (29.17)	51 (70.83)	
**Number of current medications,** mean (SD)		3.21 (1.67)	3.42 (1.59)	3.06 (1.71)	**0.03** ^a^
**Self-medication of DM medication**		55 (12.91)	36 (65.45)	19 (34.55)	**<0.001** ^a^
**Satisfaction with the healthcare system**	Not satisfied	77 (18.08)	54 (70.13)	23 (29.87)	**<0.001** ^b^
	Satisfied	349 (81.92)	126 (36.10)	223 (63.90)	
**Understanding of ongoing medication**	Understands some medication	185 (43.43)	94 (50.81)	91 (49.19)	**0.002** ^b^
	Understands all medication	189 (44.37)	62 (32.80)	127 (67.20)	
	Don’t understand	52 (12.21)	24 (46.15)	28 (53.85)	

SD = standard deviation; BDT = Bangladeshi taka (1 USD = 120 BDT)p-value <0.05 are displayed as bold^a^p- value from independent sample t-test.^b^p- value from the chi-square test.

Regarding disease-related factors, the mean duration of DM was 73.28 ± 75.67 months, with the majority taking oral medication (67.84%). Most participants took medication once daily (60.33%) and take, on average, 3.21 ± 1.67 daily. 12.91% self-medicated with DM medication, and 18.08% are not satisfied with the current healthcare. Nearly half of the participants fully understood all their medications (44.37%), and 73.71% reported good mental health status.

According to bivariate analysis, significantly higher rates of good adherence were observed among patients who live with a partner (66.67%), fully understand all their medications (67.20%), and report high satisfaction with the healthcare system (63.90%). Furthermore, a longer experience managing the disease is associated with better compliance, as patients with good adherence had a significantly longer mean duration of diabetes (86.82 months vs. 54.79 months). Conversely, severe vulnerabilities were identified around physical and mental well-being, as poor adherence was significantly more prevalent among patients experiencing poor self-perceived mental health (62.50%), living with a physical disability (56.92%), or engaging in unsupervised self-medication (65.45%) (Table 1).

In multivariate analysis, using a logistic regression model (Table 2), participants who lived with a partner had 66.4% increased odds of having good medication adherence compared to those who lived without a partner (AOR: 1.66, CI: 1.03–2.68). Higher educational status was also found to be associated with higher medication adherence. For example, participants who studied up to the undergraduate level had 2.65 times higher odds of having good adherence (AOR: 2.65, CI: 1.08–6.54). Poor mental health status was also associated with a 47% lower odds of having good medication adherence (AOR: 0.53, CI: 0.30–0.92). Participants who understood all their medication were significantly associated with 1.69 times higher odds of having good adherence than those who understood some of the medication (AOR: 1.69, CI: 1.04–2.77). For each unit increase in the number of current medications was significantly associated with a 13% lower odds of having good adherence (AOR: 0.87, CI: 0.76–1.00). Participants who self-reported the use of diabetic medication were associated with a 65% lower odds of good adherence (AOR: 0.35, CI: 0.18–0.70). Participants who were satisfied with the healthcare system reported 2.53 times higher odds of good medication adherence (AOR: 2.53, CI: 1.32–4.88).

**Table 2:** Sociodemographic and disease-related factors associated with medication adherence among the diabetic population of Bangladesh.

Characteristics	Categories	AOR	95% CI	p-value
**Sociodemographic characteristics**				
**Age** (in years)		1.02	1.00–1.05	0.091
**Sex**	Female	Ref		
	Male	1.17	0.71–1.93	0.551
**Family income** (in BDT)		1.00	1.00–1.00	0.959
**Companionship status**	Doesn’t live with a partner	Ref		**-**
	Lives with partner	1.66	1.03–2.68	**0.036**
**Educational status**	No formal education	Ref		-
	Primary	1.75	0.68–4.55	0.248
	High school	1.91	0.76–4.83	0.172
	Undergraduate	2.65	1.08–6.54	**0.034**
	Postgraduate	2.39	0.82–6.95	0.11
**Disability(visual, movement, auditory)**	No	Ref		**-**
	Yes	0.67	0.33–1.37	0.271
**Self-perceived mental health status**	Good	Ref		-
	Poor	0.53	0.30–0.92	**0.025**
**Disease-related factors**				
**Duration of DM**		1.00	1.00–1.01	**0.034**
**DM medication dosage**	Three times/day	Ref		-
	Once/day	0.46	0.19–1.09	0.078
	Twice/day	0.77	0.35–1.71	0.521
	Four times/day	0.24	0.01–6.71	0.404
**Understanding of ongoing medication**	Understands some medication	Ref		-
	Understands all medication	1.69	1.04–2.77	**0.035**
	Don’t understand	1.38	0.64–2.97	0.407
**DM medication type**	Only Oral	Ref		-
	Only Injectable	0.78	0.39–1.57	0.483
	Both Oral and Injectable	1.50	0.75–2.99	0.254
**Number of current medications**		0.87	0.76–1.00	**0.048**
**Self-medication of DM medication**	No	Ref		-
	Yes	0.35	0.18–0.70	0.003
**Satisfaction with the healthcare system**	Not satisfied	Ref		-
	Satisfied	2.53	1.32–4.88	**0.005**

AOR = Adjusted odds ratio, CI = Confidence interval;p-value <0.05 are displayed as bold

The participants also reported their recommendations regarding improving medication adherence. The majority (78.17%) of the participants suggested the need to discuss treatment options with physicians. 57.28% reported the need for frequently revising treatment regimes. Participants also indicated the need for paying more attention to mental health while prescribing (44.84%) and considering patients’ preferences (42.25%).

## Discussion

Medication adherence among DM patients plays a critical role in glycemic control, which in turn can contribute to increased morbidity and mortality. Using a validated tool (MARS-5), this cross-sectional study found that nearly half (42.25%) of the DM patients in Bangladesh have poor medication adherence, which is similar to the prevalence reported in other recent studies (48.2% and 46.3%) in Bangladesh, albeit using other validated measures (Morisky Medication Adherence Scale and Medication Compliance Questionnaire) of medication adherence [12,13]. The relatively high prevalence of poor medication adherence may reflect challenges related to healthcare access, treatment burden, limited patient counseling, and self-management practices among diabetic patients in Bangladesh. Furthermore, this is the first study in Bangladesh to comprehensively assess the personal and disease-related factors associated with medication adherence among DM patients, which is crucial to shape the future measures needed to increase medication adherence among this population.

Our study found several personal characteristics that can influence medication adherence. Having a higher educational status (AOR: 2.65, CI: 1.08–6.54), good mental health (AOR: 0.53, CI: 0.30–0.92), and a partner or companion (AOR: 1.66, CI: 1.03–2.68) can positively impact medication adherence. While higher education and mental health enable a person with a better understanding of the diseases and related complications, leading to enhanced self-care [23], a partner can provide emotional and practical support (e.g., financial support), medication information, gentle reminders, and help maintain daily routines [24,25]. A recent systematic review of 14 published papers also acknowledged family support as the most vital factor in positively influencing medication adherence [26]. Similar associations between family support and medication adherence have also been reported in other settings among patients with chronic diseases, highlighting the important role of social support in chronic disease self-management [25]. Therefore, to improve adherence, raising disease and medication-related awareness among family members can be a promising strategy, while individuals who live alone can benefit from other alternatives (e.g., digital interventions like text messages and digital companions) [27]. For example, personalized messages or individually tailored, interactive voice calls can be considered reminders, which showed higher efficacy in a prior trial on DM patients [28].

The current study also identified several disease factors, such as an increased number of medications (AOR: 0.87, CI: 0.76–1.00), an incomplete understanding of medications (AOR: 1.69, CI: 1.04–2.77), self-medication practices (AOR: 0.35, CI: 0.18–0.70), and dissatisfaction with healthcare services (AOR: 2.53, CI: 1.32–4.88), as predictors of poor medication adherence. Some of these factors are well-established determinants of medication adherence [29–31] and indicate the lack of efficient healthcare provider-receiver communication [32]. Therefore, involving healthcare providers in enhancing medication adherence can benefit LMICs like Bangladesh [32]. The current study also assessed the recommendations of study participants to improve medication adherence, which also highlighted the need for considering patients’ perspectives while prescribing medication. Furthermore, in Bangladesh, the lack of strict drug regulations and the irrational drug dispensing by the pharmacy shops enable patients to self-medicate [33] prescription medications, which can lead to serious complications, especially in diabetic patients, by causing inappropriate blood glucose levels and drug-drug interactions. Stringent government actions are required to regulate the current unregulated sales of prescription medications. Additionally, a detailed exploration of the causes of self-medication among DM patients can help determine future strategies to control this unhealthy practice.

The study has certain limitations that should be acknowledged. First, this study included a cross-sectional design, precluding any causality. Also, the use of non-probability sampling methods can influence generalizability. Additionally, since participants were recruited from health facilities, adherence observed in this study may not fully reflect adherence in the broader population. In order to mitigate the issue, participants were recruited from both government and private healthcare facilities, and the sample size was inflated to enhance population diversity. Since a standardized, diabetes-specific cut-off for the MARS-5 questionnaire has not yet been established, the cut-off used for the MARS-5 questionnaire in this analysis was derived from existing studies in different clinical cohorts [21,22]. However, it is important to note that other cut-offs for this tool also exist that may have yielded a different result on adherence. Data were self-reported by participants, including height and weight (used to calculate BMI), which may have introduced recall and measurement bias. The use of self-reported mental health has been adapted from Peterson et al. (2007) [34], due to its single-item nature, may potentially have introduced misclassification bias. However, the trained research assistants explained the questions in detail if needed and tried to validate the participants’ responses by asking follow-up questions to ensure data quality. Lastly, the study did not consider some factors (e.g., diet and counseling from medical professionals) that can influence medication adherence. In addition, the interviewer-administered nature of the survey may have introduced interviewer or social desirability bias, potentially leading to overreporting of medication adherence. To minimize the possibility of bias throughout the interview process, it was conducted in a private setting, and the participant’s comfort was taken as a priority. Finally, some important clinical, educational, and behavioral factors, such as HbA1c levels, formal diabetes education, dietary practices, and counseling, were not measured in this study and, therefore, could not be adjusted for, which may have resulted in residual confounding.

Despite these limitations, this study also had some notable strengths. This study is one of the few to examine medication non-adherence among DM patients in Bangladesh. It also identified several unique disease-related factors through a comprehensive assessment of the personal and disease-related factors that can play a pivotal role in shaping future strategies for increasing medication adherence, particularly in resource-poor settings.

## Conclusion

In sum, nearly half of the diabetic patients in Bangladesh reported poor adherence to their anti-diabetic medications. This poor adherence was significantly associated with companionship status, education, mental health, medication number, self-medication practice, and dissatisfaction with healthcare. Following that, interventions that focused on medication counseling, mental health support, and patient-centered healthcare services may play a vital role in increasing medication adherence among diabetic patients in Bangladesh.

## Supporting information

S1 File Dataset. (XLS)

S2 File Additional Information. (DOCX)
