# Supplementary material for: Factors associated with medication adherence among adult diabetic patients of Bangladesh
Source: PLoS One. 2026 Sep 17;21(9):e0343647. doi: 10.1371/journal.pone.0343647 (PMC13585251; doi:10.1371/journal.pone.0343647)
Supplement: Supplementary file 1 [file pone.0343647.s002.docx]

| ***Government hospital*** | ***Outpatient clinics*** | ***Private chambers*** |
| --- | --- | --- |
| *Chattogram Medical College Hospital (Tertiary)* | *[Zainul Haque Sikder Women's Medical College & Hospital](https://www.facebook.com/zhsikderwmch/)*  *(Urban)* | *AVE Dental Surgery and Orodontics (Urban)* |
| *Dhaka Medical College Hospital (Tertiary)* | *Chattogram Maa-O-Shishu Hospital (Urban)* | *Popular Diagnostic Centre Ltd. Chattogram (Urban)* |
| *Chattogram Medical College Dental Unit (Tertiary)* | *Institute of Applied Health Sciences(USTC) (Urban)* | *Chevron Clinical Laboratory (Pte.) Ltd. (Urban)* |
| *Bangladesh Medical University (Tertiary)* | *Chattogram General Hospital (Urban)* | *Max Hospital & Diagnostic, Chittagong (Urban)* |
| *Sir Salimullah Medical College Mitford Hospital (Tertiary)* | *Kurigram Upazilla Health Complex (Rural)* | *Trio-Dent Dental BD (Urban)* |
| *Shaheed Suhrawardy Medical College and Hospital (Tertiary)* | *Cox’s Bazar Distric Sadar Hospital (Rural)* | *Popular Diagnostic Centre (Nanupur) (Rural)* |
| *Rangamati General Hospital (Secondary)* | *Boalkhali Upazila Health Complex (Rural)* | *Janaseba Hospital (Rural)* |
| *Comilla Medical College and Hospital (Secondary)* | *Banshkhali Upazila Health Complex (Rural)* | *Drissho Dental Care (Rural)* |
| *Cox’s Bazar Medical College & Hospital(Secondary)* | *Ukhia Upazila Health Complex (Rural)* | *Chevron Clinical Laboratory, Anwara Branch (Rural)* |
| *Noakhali Medical College & Hospital(Secondary)* | *Araihazar Upazila Health Complex (Rural)* | *Upasham Medical Hall (Rural)* |

**Table S1:** List of participating Government hospitals, Outpatient clinics, and Private chambers included in the study

**Survey Questionnaire for self-medication, understanding of medications, and satisfaction with healthcare.**

*1. Which of the following drugs have you use/used for self-medication? (Self-medication has traditionally been defined as “the taking of drugs, herbs or home remedies on one’s own initiative, or on the advice of another person, without consulting a doctor)*

*☐ Diabetes medicine*

*☐ Pain killer*

*☐ Lipid-lowering drug*

*☐ Anti-ulcerant*

*☐ Blood pressure medicine*

*☐ Steroid*

*☐ Vitamins*

*☐ Iron supplements*

*☐ Calcium*

*☐ Anti-depressant/ Anxiolytics*

*☐ Anti-epileptics*

*☐ Laxatives*

*☐ Thyroid therapy*

*☐ Antibiotics*

*☐ Other ____________*

*☐ I don’t self-medicate*

*2. Do you have knowledge of the purpose, side effects, and dosing of each medication you take?*

*☐ I understand every single medication*

*☐ I understand some of them only*

*☐ I don’t understand any of my medication*

*3. Are you satisfied with your current healthcare facilities?*

*☐ No*

*☐ Yes*

**Supplementary Table 2:** Medication Adherence Questionnaire (Bengali Version)

(১ = কখনোই না, ২ = খুব কম, ৩ = মাঝে মাঝে, ৪ = প্রায়ই, ৫ = সবসময়)

| **ক্রমিক নং** | **প্রশ্ন** | কখনোই না | খুব কম | মাঝে মাঝে | প্রায়ই | সবসময় |
| --- | --- | --- | --- | --- | --- | --- |
| ১ | আমি আমার ওষুধ খেতে ভুলে যাই। | ☐ | ☐ | ☐ | ☐ | ☐ |
| ২ | আমি নিজে থেকে আমার ওষুধের মাত্রা পরিবর্তন করি। | ☐ | ☐ | ☐ | ☐ | ☐ |
| ৩ | আমি কিছু সময়ের জন্য ওষুধ খাওয়া বন্ধ করে দিই। | ☐ | ☐ | ☐ | ☐ | ☐ |
| ৪ | আমি ইচ্ছাকৃতভাবে কোনো একটি ডোজ বাদ দিই। | ☐ | ☐ | ☐ | ☐ | ☐ |
| ৫ | আমি চিকিৎসকের নির্দেশনার চেয়ে কম মাত্রায় ওষুধ গ্রহণ করি। | ☐ | ☐ | ☐ | ☐ | ☐ |
